# Headache

**Published:** 1890-02

**Authors:** 


Headache.—As a general rule, a throbbing headache, with tenderness and soreness of the scalp, can best be relieved by hot applications. Whereas, where the head feels full and "bursting," if cold be applied to the head, and the heat to the neck and spine, the effect is most agreeable.

